# A platform for the international exchange of ideas: the Israel Journal of Health Policy Research celebrates its first decade

**DOI:** 10.1186/s13584-021-00502-9

**Published:** 2021-12-15

**Authors:** Martin McKee

**Affiliations:** London School of Hygiene & Tropical Medicine, 15-17 Tavistock Place, London, WC1H 9SH UK

**Keywords:** Israel, Health policy, COVID-19

## Abstract

For ten years the Israel Journal of Health Policy Research has provided a platform for exchange of knowledge and insights on health policy. It is a unique attempt by scholars and practitioners in one small country to share their knowledge with the world and, in turn to learn from experience elsewhere. Never has this role been as important as during the COVID pandemic, a message that is very clear when we look at failings elsewhere.

I was honoured when the journal’s editors invited me to write this commentary celebrating 10 years of the journal. I have watched with great pleasure as it has gone from strength to strength under their leadership. But what should I write? Of course I could catalogue its many successes, whether measured in formal metrics or, arguably, more appropriately, in the discussions that it has stimulated about health policy in Israel and beyond [1]. However, as I reflected on this task, it became clear that there was something more profound to be said.

I must preface my comments by referring to a report published jointly by two committees of the British Parliament [2]. For several months they had been inquiring into the British government’s response to the pandemic. Their conclusions were scathing. One phrase from the report has been quoted widely. They described the response as among “the most important public health failures the United Kingdom has ever experienced.” There were many reasons, not least a profound lack of political leadership [3]. However, the committees also drew attention to the failure of ministers and their advisers to learn from experience in other countries. They noted that only one of 87 experts who attended meetings of the government’s Scientific Advisory Group for Emergencies (SAGE) was from outside the United Kingdom. Yet, from the outset, it was clear to many of us that we were to find solutions to this global crisis we would need to look to the experience of other countries. At first, these were the countries of East Asia, which had recent experience with another coronavirus, SARS [4]. They had learnt the lessons from that experience and ensured that they were prepared if something similar should happen again. The United Kingdom had not, even though it had undertaken an exercise to test its response to an imported coronavirus in 2016, something that it then tried to conceal [5]. This failure to learn from others has been a major factor in United Kingdom’s abysmal performance during the pandemic [6].

And yet, those of us outside the government’s close circle of advisers were looking elsewhere, and, especially since the end of 2020, to Israel. Given Israel’s remarkable progress in rapidly implementing a vaccination programme, it seemed obvious that there would be many lessons to learn. In the months since then, Israeli scientists have generated a wealth of knowledge on issues such as waning immunity, the role of boosters, and the frequently underplayed importance of COVID in children. And Israel also provided many important lessons that we could have learned, but too often did not, about how to address the practical challenges of implementing a vaccination programme at pace and scale [7, 8].

The United Kingdom, or more accurately England, is atypical, though sadly not unique, in its exceptionalist outlook. The United States also suffers from this problem [9]. But this insularity stands in stark contrast to the ethos that underpins this journal and which has served us so well. Uniquely (at least as far as I know) it is a journal that seeks to disseminate evidence about one country to the world and, in return, gain insights from the rest of that world. We, outside Israel, have certainly benefitted from this discourse and I believe that my Israeli counterparts have too [10].

There are so many examples of what we have learned from the pages of the journal that it is almost impossible to decide what to highlight here. We might start with more insights from the pandemic. Governments have looked at ways to increase confidence in vaccination programmes. One question is what to do in the very rare possibility that things go wrong. They will have appreciated the detailed analysis of Israel’s Vaccine Injury Compensation Law, highlighting both its strengths and its weaknesses [11]. Governments have also struggled to know what to do with schools during the pandemic. Schools, like any indoor facility, provide a setting for transmission of COVID and children do get COVID and Long COVID, with some becoming seriously ill or even dying. Yet they also suffer if they are deprived of education, potentially with life long consequences. Hence, a paper from Israel’s Multidisciplinary Academic Group on Children and Coronavirus provided many useful insights [12]. Other papers took advantage of Israel’s excellent health information resources, for example by exploring the link between social media traffic and vaccine uptake in young people [13]. In these ways, the journal has informed debate on COVID responses far beyond the borders of Israel.

Looking at the years before the pandemic, for me some of the most helpful contributions have been those that took advantage of Israel’s comprehensive health data resources, for example by helping us to understand the complexity of working with multimorbidity [14, 15], or the challenge of using routine data to adjust for risk in mental health services [16]. Then there have been the many innovations in service delivery, such as telemedicine [17], which has come into its own during the pandemic [18]. In these, as in so many other areas, the journal has provided an invaluable source of information.

So as the journal moves on to the next decade, it builds on strong foundations. And for that we can be grateful for the vision of Bruce and Avi, later joined by Steve, in making this happen.

## Data Availability

Not applicable.
